# Infections and hospital bed-days among aging adults: A five-year retrospective study in a Belgian general hospital

**DOI:** 10.3389/fmedt.2022.912469

**Published:** 2022-09-13

**Authors:** Anne-Marie De Cock, Danielle Strens, Peter Van Osta, Baudouin Standaert

**Affiliations:** ^1^ University Centre of Geriatrics, General Hospital ZNA Middelheim, Antwerpen, Belgium; ^2^ Realidad, Grimbergen, Belgium; ^3^ HEBO, Antwerpen, Belgium; ^4^ Research Unit Ethics / Patient Care, Faculty of Medicine and Life Sciences, University of Hasselt, Hasselt, Belgium

**Keywords:** aging adults, geriatrics, hospitalization, infectious disease, pulmonology

## Abstract

**Background:**

Infectious disease in aging adults (≥61 years) often occurs in combination with other health conditions leading to long hospital stays. Detailed studies on infection in aging adults investigating this problem are sparse.

**Aim:**

To quantify the effect of primary and secondary diagnosed infections on hospitalization bed-days among aging adult patients.

**Design:**

Retrospective patient-file study.

**Setting:**

Ziekenhuis Netwerk Antwerpen (ZNA) Hospital, a 1,858-bed general hospital in Belgium, with 364 beds allocated to geriatric patients.

**Data source:**

Database of hospitalized adult patients aged ≥61 years.

**Methods:**

All adult patients aged ≥61 years hospitalized on two wards, Geriatrics and Pulmonology, from 2010 to 2014 were included. Primary diagnosed infections were defined as infections known at entry to be treated first. Secondary diagnosed infections included infections known at entry but treated in parallel to primary non-infectious causes of entry, infections unknown at entry, and hospital-acquired (nosocomial) infections. Data were analyzed by patient age, gender, year, ward type, bed-days of hospitalization, infection rates, and seasonality.

**Results:**

There were 3,306 primary diagnosed infections (18%) and 14,758 secondary infections (82%) identified in the two wards combined (54.7% of all hospital stays at those 2 wards). Secondary diagnosed infections accounted for a significantly higher proportion of hospitalizations in both wards (+40% for Geriatric ward; +20% for Pulmonology ward; *p* < 0.001) and were associated with a significantly longer average hospital stay (+4 days for Geriatric ward; +5 days for Pulmonology ward; *p* < 0.001). Nosocomial infections (12% for Geriatric ward; 7% for Pulmonology ward) were associated with particularly high bed-days of hospitalization, at approximately +15 days and +12 days on Geriatric and Pulmonology wards, respectively. Both wards showed marked seasonality for respiratory infections with winter peaks.

**Conclusion:**

Real-world data showed that secondary diagnosed infections in aging adults imposed a high burden on hospital care along with longer hospital stays. This hampered bed availability during peak seasons.

## Introduction

Infectious diseases in aging adults (aged ≥61 years) often occur in the presence of multiple co-morbidities with frailty and disability (1–6). This combination of infection and co-morbidities should be assessed simultaneously to capture the whole health situation of these patients (7–11). Although this premise is established, studies reporting the total burden of infectious diseases within this broader context are sparse. Infections are often considered a minor healthcare problem and may be dominated by other chronic and non-transmissible diseases in aging adults, such as cardiovascular disease, cancer or dementia (12–14). This may result in a limited focus on infections in aging adults (15, 16). However, these diseases in this group are serious and need improved disease management (17, 18). Elderly patients are at high risk for healthcare-associated (HA) or nosocomial infections due to the age-related decline in the immune system, along with co-morbid conditions that often complicate infections and diminish the ability to treat them effectively. HA infections in elderly patients are therefore responsible for longer hospital stays, extended antibiotic therapy, significant mortality, and higher healthcare costs (19, 20). Importantly, those infections are difficult to treat, and might often be caused by antibiotic resistance (21, 22).

To explore the whole hospital infection issue amongst aging adults in greater detail, an analysis of those infections was conducted in one general hospital, the Ziekenhuis Netwerk Antwerpen (ZNA), with 1,858 beds, in Antwerp, a large city in Belgium. The aim of the study was to quantify the effect of primary and secondary diagnosed infections on hospital bed-days among aging adult patients. Two hypotheses were tested:

i) secondary diagnosed infections would be much more prevalent in elderly patients than primary diagnosed infections at hospital admission;
ii) elderly patients with primary diagnosed infections would have shorter hospital stays than elderly patients with secondary diagnosed infections.

## Material and methods

### Design

Retrospective patient-file study of hospitalized aging adults (aged ≥61 years) over a five-year period from 2010 to 2014 (23). This period was selected because at that time hospital data were collected uniformly, using the same software version.

### Setting

ZNA is an 1,858-bed hospital, with 364 beds devoted to geriatric patients. The hospital has 34 different wards where aging adults could be given care. The analysis focused on two of the wards, Geriatrics and Pulmonology, with 296 and 56 operational beds, respectively. These two wards were selected because they had a high number of aging adult patients and a high frequency of infections. The study received approval from the hospital medical ethics committee on the 14th of August 2019 (Nr 5203). All data were retrieved from the hospital database and were anonymized before any analysis was undertaken.

### Classification of infections

**Figure 1:**
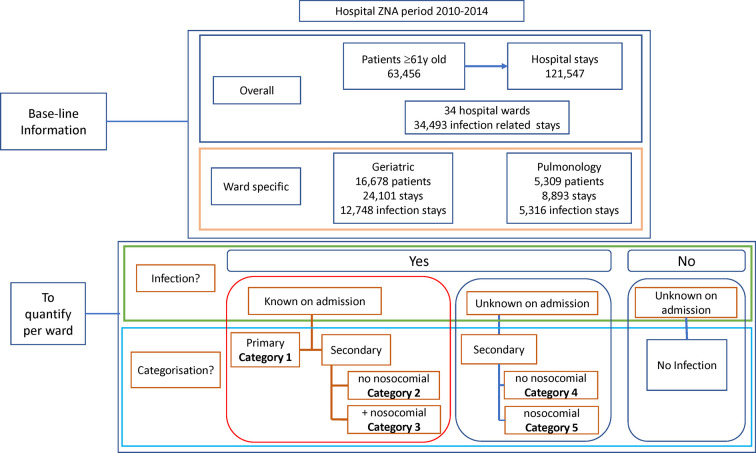
Overview of the baseline information obtained at the start of the project about the study population and the objectives of the study program.

Infections, as reported in the database, were classified into 5 categories but were first grouped into two categories of patients with infections known or unknown on admission (Figure 1), using the code Present on Admission (POA). Primary diagnosed infections were infections known on admission where the infection was the cause of hospitalization and the reason for initiating treatment. They were classified as Category 1. Secondary infections were further divided into subcategories. Category 2 were secondary diagnosed infections, known at admission and intended to be treated after treatment of the primary non-infectious disease, with no additional nosocomial infection reported. Category 3 were secondary infections that were present, known and diagnosed at hospital admission, had a treatment delay as for Category 2, and manifested a nosocomial infection acquired during the hospital stay. Category 4 were secondary infections unknown on admission with no acquired nosocomial infection. Category 5 were secondary infections unknown on admission, occurring during the hospital stay with no other infection present, and classified as nosocomial.

### Data source

The digitalized hospital database used in this study registered different diagnostic codes for each hospital stay. The first code was based on the Belgian system of All Patient Refined-Diagnosis Related Groups (APR-DRG), applied during the period 2008–2014 to collect information for the federal government using the Minimum Hospital Data system (Minimale Ziekenhuis Gegevens (MZG)) (24). The MZG system specified the ward, the bed-days of hospitalization, and the classification of secondary infection and nosocomial infection. A second code was based on the International Classification of Diseases, Ninth Revision, Clinical Modification (ICD-9-CM) (25). The ICD-9-CM code defined whether an infection was present. The third code was the Present on Admission (POA) parameter, indicating the presence and type of infection on admission.

Over the study period, the database recorded 121,547 hospital stays by 63,456 individual patients aged ≥61 years (Figure 1). Around 28% of the stays (34,493) were infection-related. Stays were concentrated in a few wards (excluding the emergency unit), as nine wards accounted for 75% of all stays. Two wards (Geriatrics and Pulmonology) had above-average infection rates for the target group and were selected for inclusion.

### Data analysis

The total number of uniquely identified patient numbers in the database defined the patient population. Each time the same patient with his/her unique code was hospitalized, an additional code was generated unique to that hospital stay. This stay code was used several times if the patient visited different wards during the stay. The number of stays divided by the number of patients over a defined period resulted in the average number of stays per patient per time unit (e.g., per year). The number of bed-days of hospital stay, or the bed occupancy, was based on the number of days billed and reported in the database.

The data from each of the two wards selected were categorized into nine five-year age groups, by gender and by the category of infection. Average bed-days of hospital stay was calculated for each infection category, and per year for trend evaluation. Seasonality was reported by calculating the number of stays in each month in a year, pooled over the five-year period and categorized as infection-related or not. Estimates of the rate of infection (overall and per infection category) per year by ward and age-group were calculated based on the total hospital bed-days occupied per year, divided by the number of infection-specific category events.

In general, data were reported per ward for the whole study period first and subsequently per year when marked differences were noted. It should be mentioned that the sum of the number of patients per type of hospital stay was not equivalent to the total number of patients overall because the same patient could have different stays during the observation period.

### Statistical analysis

Results were presented in absolute numbers per time unit, as proportions with 95% confidence intervals (95% CI), or average values with standard deviations, medians and ranges. Statistically significant differences were reported using analysis of variance (ANOVA) testing for trend results and the comparison of mean results (F-test, *p* < 0.05). Proportions were compared using Chi-square testing, *p* < 0.05.

Length of hospitalization varied between patients, and outlying values resulted in skewed distributions. Distribution slopes were therefore added using a best-fit analysis to make fair comparisons between the different groups. Accumulated probability density distributions were designed as a function of the number of bed-days of hospital stay. The median value of the slope indicated the magnitude of the difference in bed-days between groups. These distribution slopes for the hospitalization bed-days were calculated by infection category for each of the two wards. CI estimates at 95% were reported, and calculated using an add-in Excel software program, @Risk 8.2, Palisade, 2021, Raleigh, US.

All other statistical results were generated using STATA 17, 2021, Texas, US.

## Results

**Figure 2:**
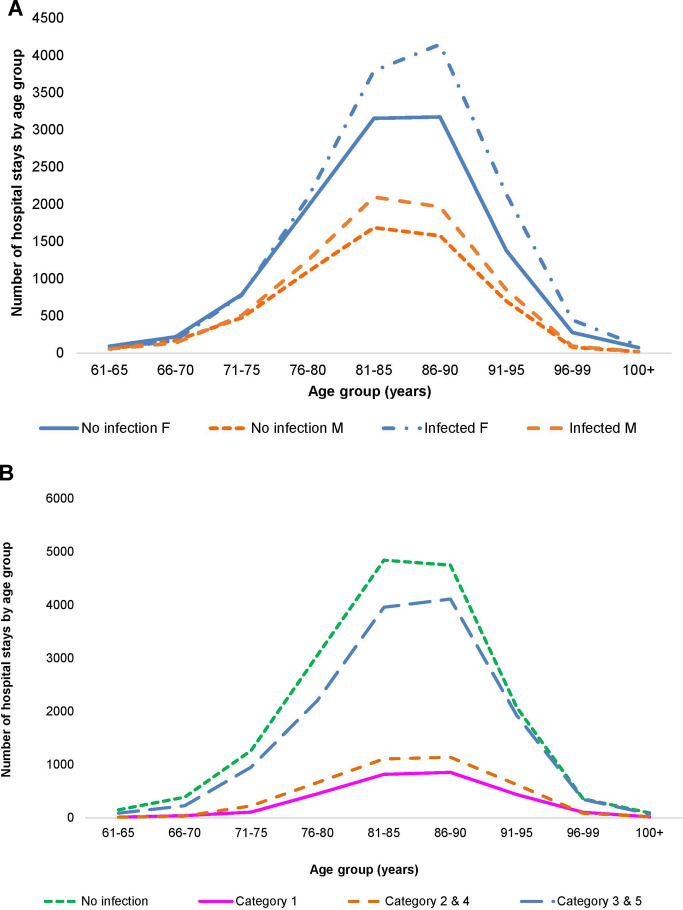
(**A**) Distribution of hospital stays (cumulative over the observation period) by age and gender in the geriatric ward. (**B**) Distribution of hospital stays (cumulative over the observation period) by infection category in the geriatric ward.

**Figure 3:**
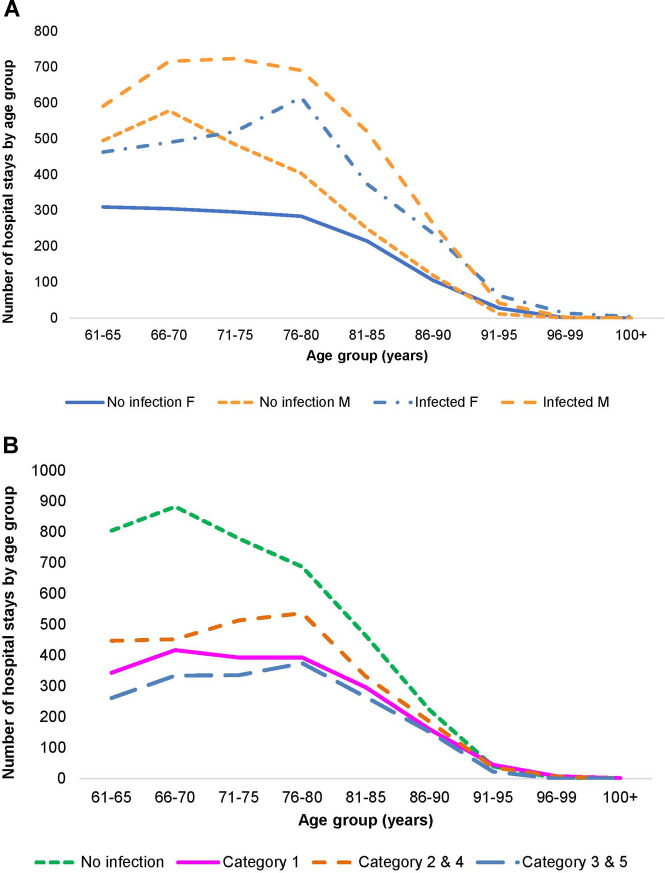
(**A**) Distribution of hospital stays (cumulative over the observation period) by age and gender in the pulmonology ward. (**B**) Distribution of hospital stays (cumulative over the observation period) by infection category in the pulmonology ward.

A total of 21,987 patients met the criteria for inclusion and the two wards had a total of 32,994 hospital stays, with Geriatric patients at 24,101 stays, and Pulmonology patients at 8,893. Infection data were available for 18,064 stays on the two wards (12,748 for Geriatrics and 5,316 for Pulmonology). Figures 2A,B, 3A,B show the distribution of the stays on the Geriatric and Pulmonology wards by age, sex and infection category. The majority (66%) of patients admitted to the Geriatric ward were female, with an age range from 61 to 106 years and an average age of 84 years (standard deviation: 6.6 years, median: 84 years). Forty-one percent of patients admitted to the Pulmonology ward were female, with an age range from 61 to 103 years and an average age of 74 years (standard deviation: 8.3 years, median: 74 years) (*t*-test of female average age in Pulmonology vs. Geriatric ward: 78.77; df = 19,584; *p* < 0.0001). There was a statistically higher rate of infection in the Pulmonology ward compared with the Geriatric ward (Tables 1, 2; 59.8% and 52.9%, respectively; Chi-Square, 124.3; df = 1; *p* < 0.0001).

**Table 1:** Hospital stays in the geriatric ward by number of patients, number of stays, bed-day of stays, and infection category (#) for the period 2010–2014.

#	Specification	Stays	%	Patients	# stay/ patient	Accumulated hospital bed days occupied	Median bed-day (days)^a^	Average bed-day (days)^a^	SD	Range (Min-Max) (days)
	**Overall**									
	Total patient population	**24,101**		16,678	1.45	473,292	16	19.88	15.17	1–226
	No infection	**11,353**	**47.1%**	9,196	1.23	199,856	15	17.76	17.76	1–183
	Infection	**12,748**	**52.9%**	9,814	1.30	273,436	18	21.61	16.33	1–226
	Known at admission	3,690	28.9%	3,325	1.11	61,355	13	16.82	13.65	1–149
4 + 5	Unknown at admission	9,058	71.1%	7,568	1.20	212,081	20	23.56	16.92	1–226
	**Infection known at admission**	**3,690**	**15.3%**							
1	Primary	1,560	42.3%	1,434	1.09	21,799	12	14.14	10.18	1–98
2	Secondary only	1,768	47.9%	1,587	1.11	29,061	14	16.54	12.30	1–96
3	Secondary + nosocomial	362	9.8%	357	1.01	10,495	24	30.06	22.86	1–149
2 + 3	Sum secondary known	2,130	57.7%	1,891	1.13	39,556	15	17.74	15.59	1–149
	**Infection unknown at admission**	**9,058**	**37.6%**							
4	Secondary only	6,521	72.0%	5,619	1.16	137,837	18	21.26	14.82	1–217
5	Nosocomial only	2,537	28.0%	2,428	1.04	74,244	26	29.45	20.22	1–226

Max, maximum; Min, minimum; MZG, Minimale Ziekenhuis Gegevens; SD: Standard Deviation.

^a^ Based on “G” code of MZG index.

**Table 2:** Hospital stays in the pulmonology ward by number of patients, number of stays, bed-day of stays, and infection category (#) for the period 2010–2014.

#	Specification	Stays	%	Patients	# stay/patient	Accumulated hospital bed days occupied	Median bed-day (days)^a^	Average bed-day (days)^a^	SD	Range (Min-Max) (days)
	**Overall**									
	Total patient population	**8,893**		5,309	1.68	75,683	6	8.51	9.35	1–153
	No infection	**3,577**	**40.2%**	2,434	1.47	18,523	2	5.17	6.58	1- 64
	Infection	**5,316**	**59.8%**	3,559	1.49	57,160	8	10.89	10.24	1–153
	Known at admission	3,785	71.2%	2,617	1.45	39,227	8	10.47	9.08	1–124
4 + 5	Unknown at admission	1,531	28.8%	1,285	1.19	17,933	9	11.94	12.61	1–90
	**Infection known at admission**	**3,785**	**42.6%**							
1	Primary	1,746	46.1%	1,395	1.25	14,860	7	8.51	6.83	1–73
2	Secondary only	1,790	47.3%	1,328	1.35	19,300	9	10.78	8.48	1–124
3	Secondary + nosocomial	249	6.6%	241	1.03	5,067	17	20.34	16.81	1–90
2 + 3	Sum secondary	2,039	53.9%	1,504	1.36	24,367	9	11.95	10.40	1–124
	**Infection unknown at admission**	**1,531**	**17.2%**							
4	Secondary only	1,192	77.9%	1,009	1.18	11,808	8	9.90	9.68	1–75
5	Nosocomial only	339	22.1%	332	1.02	6,125	13	18.07	18.32	1–153

Max, maximum; Min, minimum; MZG, Minimale Ziekenhuis Gegevens; SD, Standard Deviation.

^a^ Based on “D” code of MZG index.

**Figure 4:**
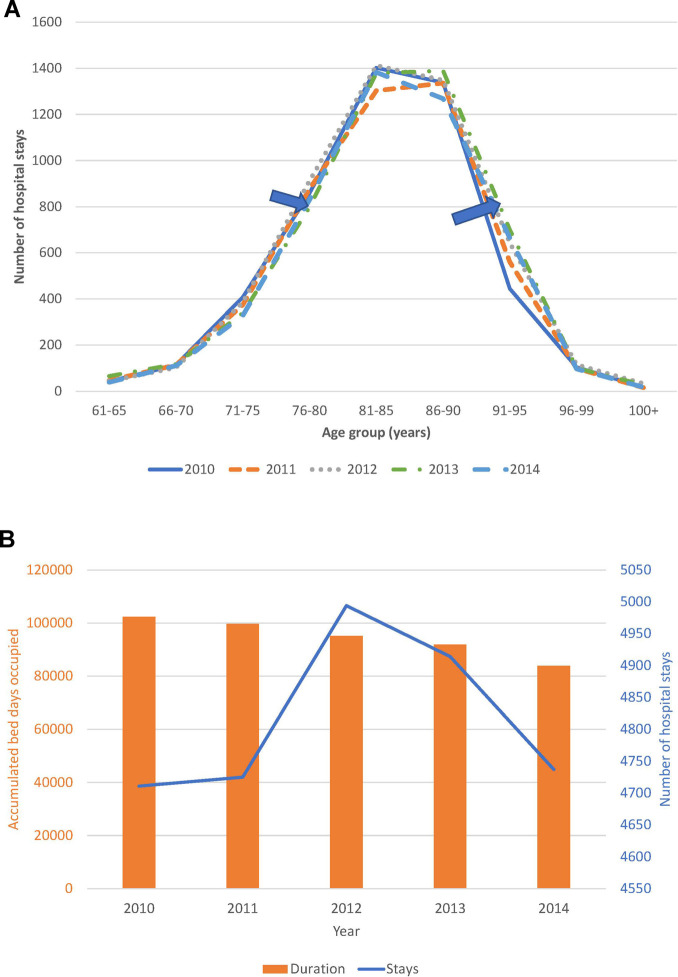
(**A**) Time trend in the age distribution per year for the geriatric ward. (**B**) Time trend for accumulated hospital bed-days (bars) with hospital stays (line) per year in the geriatric ward.

**Figure 5:**
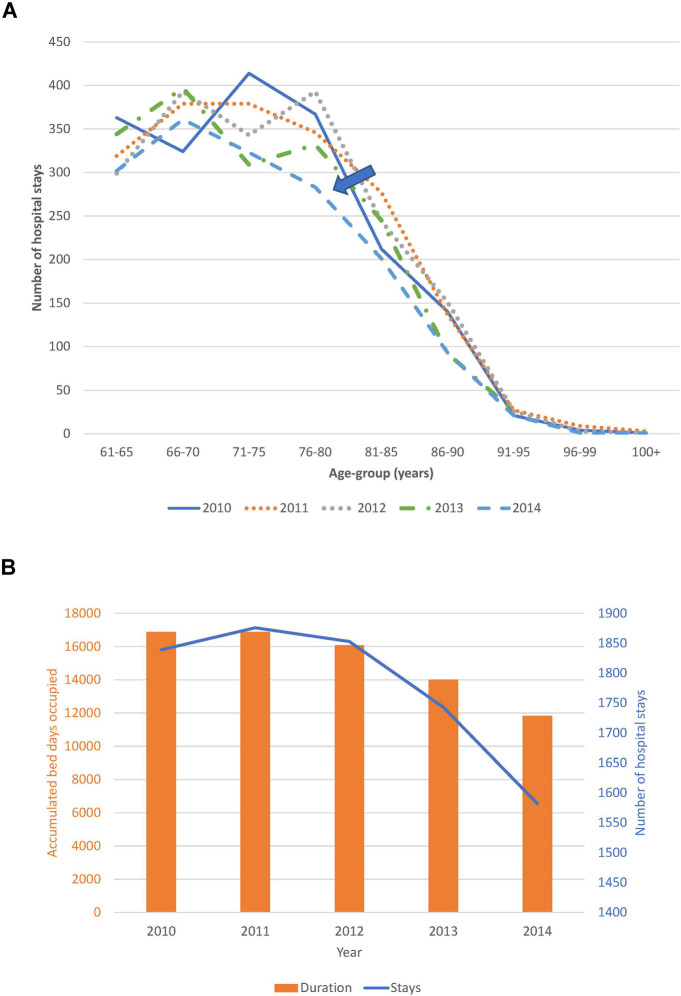
(**A**) Time trend in the age distribution per year for the pulmonology ward. (**B**) Time trend for accumulated hospital bed-days (bars) with hospital stays (line) per year in the pulmonology ward.

Trend analysis of changes in the age distribution of the patient population over time, and hospital stays and bed-days per year, are reported in Figure 4A,B for the Geriatric ward and in Figure 5A,B for the Pulmonology ward. The age structure of the patient population in the Geriatric ward shifted slightly to the right over time (blue arrows), but the accumulated hospital bed occupancy (bed-days occupied) per year decreased from 102,401 days in 2010 to 83,835 days in 2014 (−18%), while hospital stays per year peaked in 2012 at 4,994 stays but remained approximately stable between the years 2010 and 2014 at around 4,720 stays per year (+0.5%). This was in contrast with the Pulmonology ward, where all parameters decreased from 2010 to 2014 (age distribution (−14%; 1,586/1,846), bed-days (−30%; 11,823/16,885), and hospital stays (−14%; 1,582/1,839).

Table 1 outlines the number of hospital stays on the Geriatric ward for the whole study period, with the average overall bed-days by infection category. A total of 12,748 of the 24,101 hospital stays on the Geriatric ward were infection-related (52.9%; 95% CI, ±0.6%). It should be noted that the range of duration (last column in Tables 1, 2) had a minimum value of 1 day because any patient of the group hospitalized for a minimum of 1 day for a check-up or for an intravenous drug administration defined the lowest value of the range of the duration for the group.

Primary infections accounted for 6.5% of all stays (1,560/24,101), and secondary infections unknown at entry for 37.6% (9,058/24,101). The number of hospital stays per patient indicated how often the same patients may have returned. This was low for primary infections and nosocomial infections, but not for other categories of secondary infections.

Of infections known at admission, primary infections (Category 1) represented 42.3% (1,560/3,690), secondary infections identified on admission with no nosocomial infection (Category 2) represented 47.9% (1,768/3,690) and known infections with additional nosocomial infection (Category 3) was a small group, accounting for 9.8%, (362/3,690). Of secondary infections unknown on admission, those with no nosocomial infection (Category 4) represented 72% (6,521/9,058), and nosocomial infections alone (Category 5) represented 28% (2,537/9,058).

Hospital stays with unknown secondary infections were much more common (9,058; 71.0%) than known secondary infections (2,130; 16.7%) or primary infections (1,560; 12.3%) (Chi-square: 223.14; *p* < 0.0001). The median number of bed-days of hospitalization was 18 days across all infection categories, with higher median bed-days of up to 26 days for nosocomial infections (Table 1).

**Figure 6:**
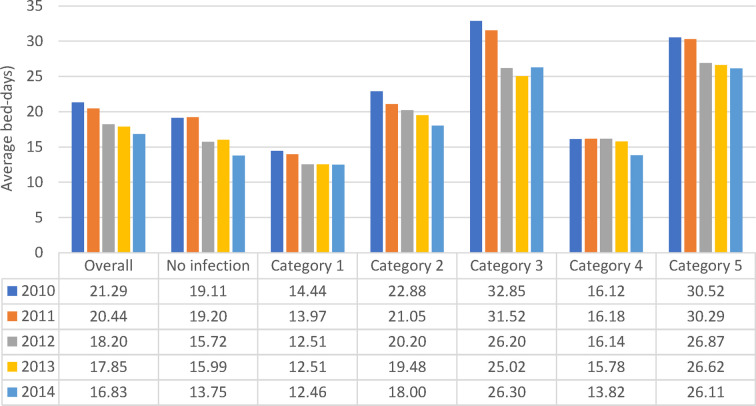
Average hospital bed-days by infection category over the years of observation for the geriatric ward.

More infections occurred in women (8,418/15,900) than in men (4,319/8,181) (Figure 2A), but the percentage with infections was similar, around 53% (Chi-square: 0.22; *p* = 0.8248). A comparison between 2010 and 2014 showed a significantly larger increase in infections in men aged ≥80 years (increase of 107 cases; (+32%; 538 to 645) and 336 additional stays (1,187 to 1,523)) than women aged ≥80 years (increase of 12 cases; (7%; 1,291 to 1,303) and 164 additional stays (2,692 to 2,856)) (Chi-square: 6.06; *p* < 0.0001). The average bed-days of hospital stay on the Geriatric ward decreased overall from 21.29 days in 2010 to 16.83 days in 2014 (ANOVA, F-value: 58.92, *p* < 0.0001) (Figure 6).

All nosocomial infections together accounted for 12% of Geriatric ward stays ((362 + 2,537)/24,101). Across the study period, the average bed-days of nosocomial infections was approximately 15 days longer than primary infections. However, the average bed-days of hospital stay decreased over time for nosocomial infections, from 32.61 days in 2010 to 26.27 days in 2014 (ANOVA: F-value: 13.64; *p* < 0.0001) (Figure 6). The absolute difference between the shortest infection average stay and the nosocomial infection average stay improved only slightly over time, by 2 days, from 16 days difference in 2010 to 14 days difference in 2014.

**Figure 7:**
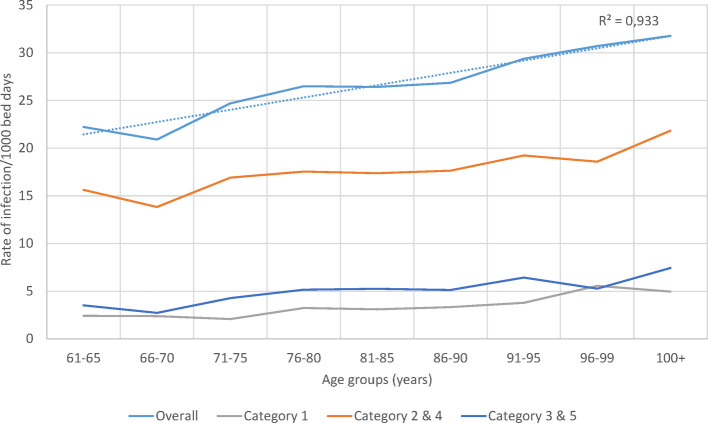
Infection rate overall and for the different infection categories per 1,000 bed-days occupied during the observation period (2010–2014) by age group in the geriatric ward.

The rate of infection by age group and category over the whole observation period is presented in Figure 7. A significant linear increase in infection rate by age group for overall infection, linked to secondary infection (Categories 2 / 4), was seen.

Although annual bed-days and annual hospital stays both decreased over time in the Pulmonology ward (Figure 5B), this was not so clearly seen in the Geriatrics ward where annual stays peaked in the middle of the observation period while annual bed-days decreased slightly over the period (Figure 4B). The remaining high stay may be explained by the gradual infection rate increase by age seen in Figure 7, linked to the aging population demographic by year (Figure 4A).

**Figure 8:**
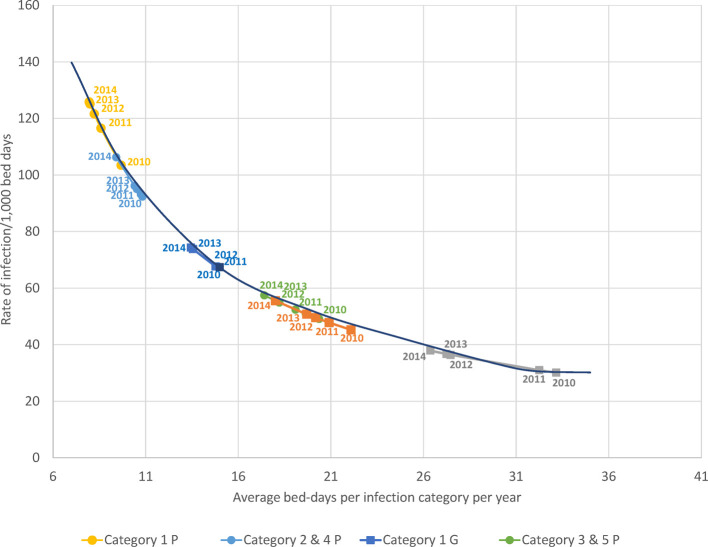
The observed relationship between average bed-days per stay by infection category measured per year and the rate of infection for each category in the geriatric (squares) and pulmonology wards (circles). G, Geriatric; *P*, Pulmonology.

Figure 8 shows observed data per year for the average bed-days by infection category linked to the resulting rate of infection measured. The pattern of lower bed-days linked to higher rates was observed for all infection categories in both wards. A regression equation was constructed to predict the numbers outside the reported data (R^2 ^= 0.99) and plotted in Figure 8 as “simulated equation”. High average hospitalization bed-days, as seen in 2010, were linked to lower rates of infection observed because of the limitations of bed availability in the ward, while lower average bed-days as seen in 2014 were linked to higher rates of infection.

Finally, based on the data reported in Table 1, an excess in hospital bed-days was calculated for secondary (Categories 2 / 4) and nosocomial infections (Categories 3 / 5) taking the average bed-days of a primary infection as a reference. The excess number of hospital bed-days for secondary infections and nosocomial infections was 54% (+51,070 bed-days) and 46% (+44,229 bed-days), respectively, compared with primary infections.

As outlined in Table 2, data for Pulmonology ward patients differed from Geriatric ward patients. Of the 8,893 total Pulmonology ward hospital stays, 5,316 (59.8%; 95% CI, ±1%) were infection-related, a higher proportion than in the Geriatric ward. The data on hospital stays showed that primary infections (Category 1) accounted for 19.6% of all stays (1,746/8,893), and secondary infections unknown at admission accounted for only 17.2% (1,531/8,893) (Figure 3B). Nosocomial infections (Categories 3 / 5) accounted together for 6.6% ((249 + 339)/8,893). The median number of bed-days of hospitalization across all infection categories in the Pulmonology ward was 8 days, considerably lower than in the Geriatrics ward.

Of all the registered hospital infection stays there was a higher proportion of primary infections on the Pulmonology ward than on the Geriatric ward (32.8% (1,746/5,316) vs. 12.2%, Chi-square: 32.66; *p* < 0.0001), and a lower proportion of secondary infections (67.2% (3,570/5,316) vs. 87.8%, Chi-square: 32.66; *p* < 0.0001). There was also a lower proportion of nosocomial infections in the Pulmonology ward than in the Geriatric ward (11.1% (588/5,316) vs. 22.7%, Chi-square: 18.11; *p* < 0.0001). The average bed-days of hospital stays in the Pulmonology ward decreased from 9.2 days in 2010 to 7.5 days in 2014 (ANOVA, F-value = 9.84, *p* < 0.0001). The average bed-days of hospital stays in the Pulmonology ward also decreased over time for nosocomial infections (Category 5), from 21.80 days in 2010 to 18.12 days in 2014. However, the decrease was not statistically significant (ANOVA, F-value = 0.73; *p* = 0.57). There was also an overall decrease in hospital stays among younger men and women patients. The number of stays decreased by 123 days (25%) between 2010 and 2014 for women and by 139 days (22%) for men. Over the study period, nosocomial infections in the Pulmonology ward were associated with approximately 12 days higher average bed-days of hospitalization than primary infections (Table 2). Finally, the rate of infection was stable across the different age-groups considered (around 7 infections per 100 bed-days occupied in the group aged ≤90 years old, increasing to much higher rates for the oldest age group (>25 for the group aged ≥91 years). Figure 8 reports for the Pulmonology ward the observed relationship between average bed-days by infection category per year and the rate of infection. Those data (circles) are obviously more to the left of the curve, as compared with the Geriatric ward (squares). The excess hospital bed-days for secondary and nosocomial infections were in the opposite direction from the Geriatric ward, more for nosocomial infections (52%; +6,188 bed days) than for secondary infections (48%; +5,729).

**Figure 9:**
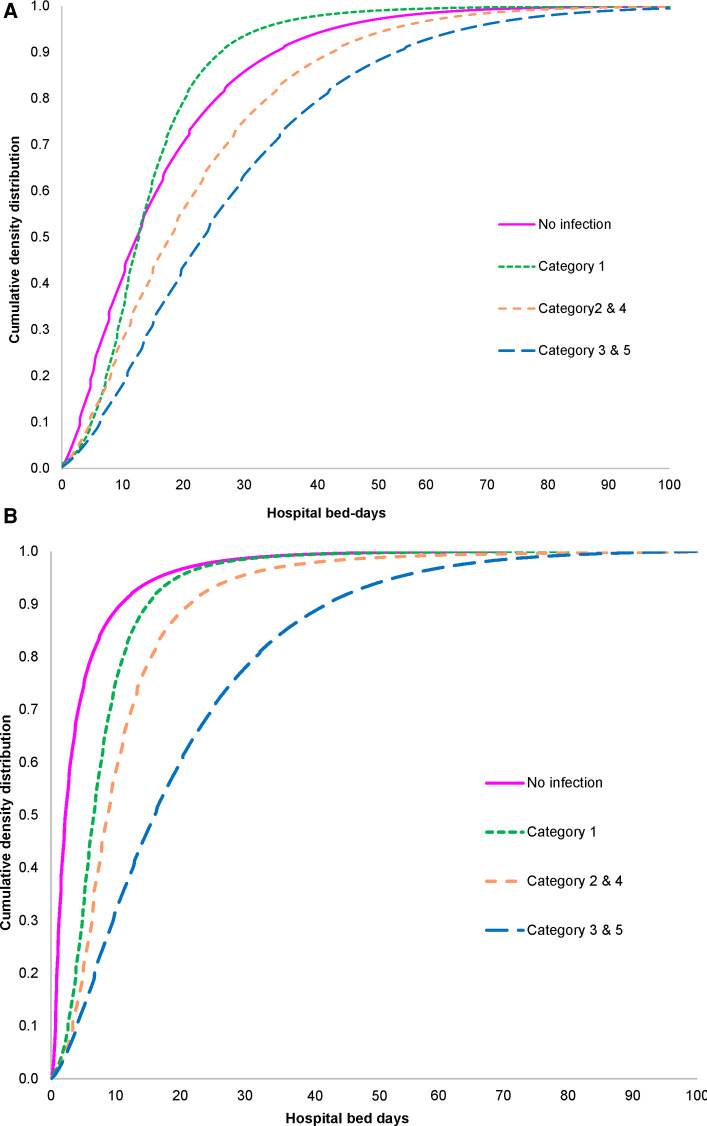
(**A**) Slope of hospitalization bed-days for different infection categories in the geriatric ward. (**B**) Slope of hospitalization bed-days for different infection categories in the pulmonology ward.

Adding distribution slopes using a best-fit analysis to make fair comparisons between different subgroups changed the slope of hospitalization bed-days. Figure 9A,B show the slope distributions of accumulated probabilities for different infection categories for the Geriatric ward and the Pulmonology ward, respectively. In the Geriatric ward, the curve for primary infection was furthest to the left (Figure 9A), indicating that number of hospitalization bed-days was generally lower when a known infection at entry, the cause of hospitalization for treatment, was the primary diagnosis. Known secondary infections increased the bed-days, and nosocomial infections extended the length of hospital stays considerably.

Hospital bed-days on the Pulmonology ward (Figure 9B) were generally lower than on the Geriatric ward and the curves for no infection, primary infection and secondary infection were clustered together. Nosocomial infections were associated with a considerably longer stay than any other infection (Figure 9B). The curves for the Pulmonology ward reached the top earlier than the curves for the Geriatric ward, reflecting the longer maximum stay on the Geriatric ward of 226 days (Table 1), compared with 153 days on the Pulmonology ward (Table 2).

**Figure 10:**
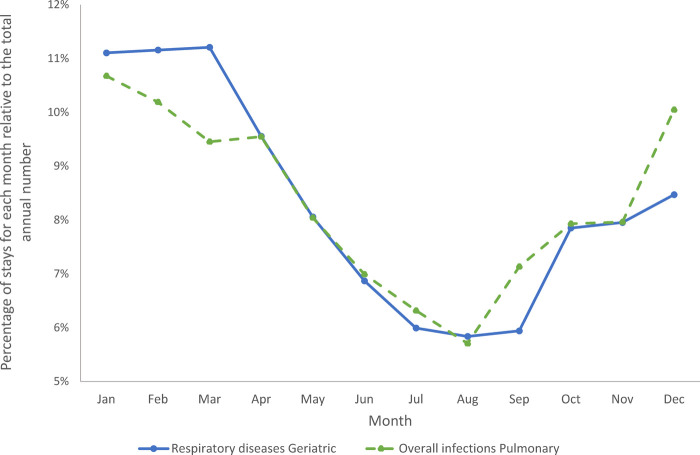
Cumulative seasonality trends for overall infections in the pulmonary ward and for respiratory diseases in the geriatric ward.

Figures 10 shows the percentage of stays in each month for the Geriatric ward and the Pulmonology ward, respectively. The most prevalent infectious diseases were respiratory infections on both wards. A full description of the infection presentations per category and per ward is presented in Supplementary Materials. It should be mentioned that the infection profile was different in the groups of Category 1 (primary infection) vs. Categories 2 / 4 (secondary infection only), and Categories 3 / 5 (nosocomial infection). However, as expected, overall infections in the Pulmonology ward showed a strong seasonal pattern (Figure 10), with infections peaking in the winter months (95% CI between 5% and 8%). Respiratory diseases in the Geriatric ward showed a similar but more pronounced seasonal pattern (Figure 10) (95% CI between 3% and 5%).

## Discussion

This retrospective study provides real-world data on the pattern of infections in hospitalized aging adults (≥61 years) over a five-year period in a 1,858-bed city hospital in Belgium with 364 geriatric beds. The study particularly considered the type of infection, defined by its time of diagnosis, linked to the duration of hospital care. The study did not look at differences in infectious diseases linked to their severity levels but had a focus on presence or absence of infection. The results confirmed our two initial hypotheses, as there were more secondary than primary infections in both wards studied, and the average number of bed-days of hospitalization was significantly higher for secondary infections. This was particularly true for nosocomial infections. The results showing excess bed-days for secondary infections compared with primary infections illustrated the size of the resulting healthcare burden. The extended hospital stays may also have consequences for recovery. The occurrence and severity of secondary infections were linked to age, the presence of co-morbidities and frailty (26). Although average hospitalization bed-days across all infections decreased over the study period, possibly reflecting changes in infection management, the additional hospitalization bed-days associated with nosocomial infection compared with primary infection showed little change over time (27). This suggests that the complexity of treatment and the protracted recovery for patients with nosocomial infections may be related more to the patient profile than to the infection management policy. This potential explanation will be the subject of a further evaluation of the data, exploring the link between infection type and its cause and patients' health condition and other demographic characteristics. It may prove challenging to address those links as demographic changes further increase the average age of hospitalized patients. It may be possible that improvements in ward organization could help recovery by offering more focused bedside care and interventions to avoid sarcopenia, delirium, and frailty (2).

The point to make about those study results related to health technology assessment is that working with an average value for the duration of hospital stay of an infection may undervalue the true number. The latter could be much higher related to the level of heterogeneity present in the data for demographic criteria and health conditions with the presence or absence of co-morbidities. Infections in hospital care cannot be summarized into one number, but it needs a detailed evaluation so that the reality of the problem may appear.

Meanwhile, in this analysis the calculation of infection rate estimates were explored amongst the patients under study. Those rates were approximate estimates of the real infection rate in the population, because the data were influenced by limiting factors such as bed-day management rules, bed capacity, or seasonality of the infection causing peaks. It was interesting to note (as shown in Figure 7) that the overall infection rate in the Geriatric ward increased with age, which could be the result of secondary and nosocomial or HA infections that correlated well with the overall increase.

Analyzing combined data from the Geriatric and Pulmonology wards in more depth indicated that predictions can be made about incoming rates of infection from the reduction in hospitalization bed-days by infection category and ward type (Figure 8). This could be informative for hospital management and may allow adjustment of beds needed by ward type depending on infection category. The sustained reduction in hospital stays linked to the reduction in bed-days occupied in the Pulmonology ward (Figure 5B), could be explained by elements such as a real measured reduction in infections in this age group, or shifts of treatment to outpatient care or other wards such as the Geriatric ward. The same profile did not appear in the Geriatric ward, indicating that the Geriatric ward was operating during the study period at its limits of maximum capacity.

Nosocomial or HA infections were complex to handle and needed more dedicated nursing and medical supervision, increasing the burden on hospital resources (28). The seasonal pattern in infections observed on both wards had the potential to place severe pressure on bed occupancy during winter peak periods, with potential challenges for infection management. Under-capacity and high bed occupancy may affect quality of care, as has previously been observed in pediatric wards (29), and may also place healthcare professionals at higher risk of infection. Seasonal pressure could be mitigated by seasonal reorganizations on wards treating aging adults, adapting the number of staff and hospital beds during winter to improve patient care, improve the work environment and decrease burnout risk in healthcare professionals.

The high numbers and long hospital stays of patients with secondary infections could be an important source of in-hospital infection, potentially increasing treatment resistance over time (30–32). Current healthcare structures bring large numbers of patients, who may be vulnerable because of other health conditions and age-related immune decline, together in dense environments. This can dramatically increase infection transmission. The present results showed that the pool of secondary infections was large, around 40% of the activity in Pulmonology and over 45% in Geriatrics. Strategies are needed to reduce the problem. In addition to better prevention, safer procedures, better diagnostics or better testing, new construction concepts for high-risk wards might reduce the risk of nosocomial infection. These elements are currently being evaluated by the VITAL-IMI project, a research program on aging adults and infectious diseases in Europe sponsored by Horizon 2020 and the European Federation of Pharmaceutical Industries and Associations (EFPIA) (33).

Although both wards experienced high rates of infection, the Pulmonology ward had a lower number of unknown infections at entry and a more pronounced relative difference in length of stay between patients with nosocomial infections and the other groups than the Geriatric ward. The Geriatric ward had a stronger link than the Pulmonology ward to the Emergency unit, where screening for infection should be recommended. The decrease in Pulmonology stays and the increase in Geriatrics stays observed over the study period may indicate a potential shift from Pulmonology to Geriatrics in this period.

The data for this study were uniformly collected from 2010 to 2014 throughout the period of observation, to limit the potential for bias resulting from new rules of registration or new ways to manage healthcare programs. Changes in the software programs effectively occurred in the years following the evaluation period, with the removal of the MZAG-code for nosocomial infections and a shift from ICD-9 to ICD-10 codes. Changes in software registries are now happening frequently, as computer systems and programs are becoming more powerful and capable of embracing more data, improving the potential for data collection and evaluation (34). Furthermore, this study was conducted on data from patients aged ≥61 years admitted to a single large general hospital in Belgium. Therefore, although the results may highlight issues that may be important in other hospitals with similar settings, they may not be applicable to other patient populations in different countries and different settings. More recent data could be collected and analyzed to obtain the same quality in registration and coding to update the analysis carried out in this study.

This study used hospital database data to demonstrate that amongst hospitalized aging adults secondary infections were more common than primary infections and were associated with longer hospital stays. However, the secondary group of infections are often not reported in official statistics. Only primary diagnosed and nosocomial infections are shown, resulting in a clear underreporting of the true healthcare problem of infections in hospital care. There is therefore a need to report those data and to develop improved healthcare solutions for management of secondary infections in aging adults (35, 36).

## Conclusion

The analysis method developed and the results obtained from this study should be helpful for healthcare authorities and decision-makers to better understand the full potential burden of infections in hospitalized aging adults and its relationship to the problem of bed availability. This study may provide baseline figures from a period before the COVID pandemic, and may also be of value for health economic evaluations of proposed new intervention options. In addition, government, private and international bodies working on providing healthcare may find the information useful to determine management and future strategies to tackle secondary diagnosed infections in aging adults. For many of the infections seen in this environment, preventative measures such as improved hygiene in hospital care and/or better implementation of vaccination programs, could substantially reduce the critical healthcare issue of extended hospital stays.

## Data Availability

The original contributions presented in the study are included in the article/Supplementary Material, further inquiries can be directed to the corresponding author/s.

## References

[1] Beard JR Bloom DE. Towards a comprehensive public health response to population ageing. Lancet. (2015) 385(9968):658–61. 10.1016/S0140-6736(14)61461-6 25468151 PMC4663973

[2] Fried LP Ferrucci L Darer J Williamson JD Anderson G. Untangling the concepts of disability, frailty, and comorbidity: implications for improved targeting and care. J Gerontol A Biol Sci Med Sci. (2004) 59(3):255–63. 10.1093/gerona/59.3.m255 15031310

[3] Pilotto A Veronese N Daragjati J Cruz-Jentoft AJ Polidori MC Mattace-Raso F Using the multidimensional prognostic index to predict clinical outcomes of hospitalized older persons: a prospective, multicenter, international study. J Gerontol A Biol Sci Med Sci. (2019) 74(10):1643–9. 10.1093/gerona/gly239 30329033 PMC6940980

[4] Schoevaerdts D Sibille FX Gavazzi G. Infections in the older population: what do we know? Aging Clin Exp Res. (2021) 33(3):689–701. 10.1007/s40520-019-01375-4 31656032

[5] Kristensen M van Lier A Eilers R McDonald SA Opstelten W van der Maas N Burden of four vaccine preventable diseases in older adults. Vaccine. (2016) 34(7):942–9. 10.1016/j.vaccine.2015.12.052 26752065

[6] Norman DC. Clinical features of infection in older adults. Clin Geriatr Med. (2016) 32(3):433–41. 10.1016/j.cger.2016.02.005 27394015

[7] Cesari M Marzetti E Canevelli M Guaraldi G. Geriatric syndromes: how to treat. Virulence. (2017) 8(5):577–85. 10.1080/21505594.2016.1219445 27540686 PMC5538337

[8] Esper AM Moss M Lewis CA Nisbet R Mannino DM Martin GS. The role of infection and comorbidity: factors that influence disparities in sepsis. Crit Care Med. (2006) 34(10):2576–82. 10.1097/01.CCM.0000239114.50519.0E 16915108 PMC3926300

[9] Maltezou HC Raftopoulos V Vorou R Papadima K Mellou K Spanakis N Association between upper respiratory tract viral load, comorbidities, disease severity, and outcome of patients with sars-Cov-2 infection. J Infect Dis. (2021) 223(7):1132–8. 10.1093/infdis/jiaa804 33388780 PMC7798974

[10] Cilloniz C Rodriguez-Hurtado D Torres A. Characteristics and management of community-acquired pneumonia in the era of global aging. Med Sci (Basel). (2018) 6(2), 35. 10.3390/medsci6020035 PMC6024853 29710871

[11] Yoshikawa TT Norman DC. Geriatric infectious diseases: current concepts on diagnosis and management. J Am Geriatr Soc. (2017) 65(3):631–41. 10.1111/jgs.14731 28140454

[12] Mouton CP Bazaldua OV Pierce B Espino DV. Common infections in older adults. Am Fam Physician. (2001) 63(2):257–68. 11201692

[13] Pinner RW Teutsch SM Simonsen L Klug LA Graber JM Clarke MJ Trends in infectious diseases mortality in the United States. JAMA. (1996) 275(3):189–93. 10.1001/jama.1996.03530270029027 8604170

[14] Yoshikawa TT. Epidemiology and unique aspects of aging and infectious diseases. Clin Infect Dis. (2000) 30(6):931–3. 10.1086/313792 10880303

[15] Glynn JR Moss PAH. Systematic analysis of infectious disease outcomes by age shows lowest severity in school-age children. Sci Data. (2020) 7(1):329. 10.1038/s41597-020-00668-y 33057040 PMC7566589

[16] Cassini A Colzani E Pini A Mangen MJ Plass D McDonald SA Impact of infectious diseases on population health using incidence-based disability-adjusted life years (Dalys): results from the burden of communicable diseases in Europe study, European union and European economic area countries, 2009 to 2013. Euro Surveill. (2018) 23(16):17-00454. 10.2807/1560-7917.ES.2018.23.16.17-00454 PMC5915974 29692315

[17] Schneider EL. Infectious diseases in the elderly. Ann Intern Med. (1983) 98(3):395–400. 10.7326/0003-4819-98-3-395 6830081

[18] De Foor J Senterre C Leclercq P Martins D Pirson M. Profile of hospitalised elderly patients in Belgium—analysis of factors affecting hospital costs. J Econ Ageing. (2020) 15:100209. 10.1016/j.jeoa.2019.100209

[19] Cristina ML Spagnolo AM Giribone L Demartini A Sartini M. Epidemiology and prevention of healthcare-associated infections in geriatric patients: a narrative review. Int J Environ Res Public Health. (2021) 18(10):5333. 10.3390/ijerph18105333 34067797 PMC8156303

[20] Suetens C Latour K Karki T Ricchizzi E Kinross P Moro ML Prevalence of healthcare-associated infections, estimated incidence and composite antimicrobial resistance Index in acute care hospitals and long-term care facilities: results from two European point prevalence surveys, 2016 to 2017. Euro Surveill. (2018) 23(46):pii=1800516. 10.2807/1560-7917.ES.2018.23.46.1800516 PMC6247459 30458912

[21] Vandael E Latour K Goossens H Magerman K Drapier N Catry B Point prevalence survey of antimicrobial use and healthcare-associated infections in Belgian acute care hospitals: results of the global-PPS and ECDC-PPS 2017. Antimicrob Resist Infect Control. (2020) 9(1):13. 10.1186/s13756-019-0663-7 31956402 PMC6958935

[22] Cassini A Hogberg LD Plachouras D Quattrocchi A Hoxha A Simonsen GS Attributable deaths and disability-adjusted life-years caused by infections with antibiotic-resistant Bacteria in the eu and the European economic area in 2015: a population-level modelling analysis. Lancet Infect Dis. (2019) 19(1):56–66. 10.1016/S1473-3099(18)30605-4 30409683 PMC6300481

[23] Facon P. Blikvanger Gezondheidszorg Algemene Ziekenhuizen. (2019) [cited May 5, 2021]. Available from: https://www.health.belgium.be/sites/default/files/uploads/fields/fpshealth_theme_file/blikvanger_gezondheidszorg_az_v10.pdf

[24] MZG. Bijkomende Informatie over Bedindexen, Verpleegeenheden En Verpleegkundige Zorgperioden. (2019) [cited May 5, 2021]. Available from: https://www.health.belgium.be/sites/default/files/uploads/fields/fpshealth_theme_file/bijkomende_informatie_over_bedindexen_verpleegeenheden_en_zorgperioden_2019_12_0.pdf

[25] Institute FP. Handboek Icd-9-Cm-Codering (2013). [cited May 5, 2021]. Available from: https://www.health.belgium.be/sites/default/files/uploads/fields/fpshealth_theme_file/codeerhandboek_icd-9-cm_2013_0.pdf

[26] Fried LP. Epidemiology of aging. Epidemiol Rev. (2000) 22(1):95–106. 10.1093/oxfordjournals.epirev.a018031 10939013

[27] Inweregbu KD Jayshree Pittard A. Nosocomial infections. Br J Anaesth. (2005) 5(1):14–7. 10.1093/bjaceaccp/mki006

[28] Stubbiefield H. What Are Nosocomial Infections? Healthline. (2017) [cited May 5, 2021]. Available from: https://www.healthline.com/health/hospital-acquired-nosocomial-infections

[29] Standaert B Alwan A Strens D Raes M Postma MJ. Improvement in Hospital Quality of Care (Qoc) after the introduction of rotavirus vaccination: an evaluation study in Belgium. Hum Vaccin Immunother. (2015) 11(9):2266–73. 10.1080/21645515.2015.1029212 25902371 PMC4635727

[30] Reed D Kemmerly SA. Infection control and prevention: a review of hospital-acquired infections and the economic implications. Ochsner J. (2009) 9(1):27–31. 21603406 PMC3096239

[31] Sydnor ER Perl TM. Hospital epidemiology and infection control in acute-care settings. Clin Microbiol Rev. (2011) 24(1):141–73. 10.1128/CMR.00027-10 21233510 PMC3021207

[32] Vandael E Boudewijn C Latour K. Point Prevalence Study of Healthcare Associated Infections and Antimicrobial Use in Belgian Acute Care Hospitals: Results of the ECDC PPS 2017. Sciensano (2018) D/2018/14.440/37.

[33] Van Baarle D Bollaerts K Del Giudice G Lockhart S Luxemburger C Postma MJ Preventing infectious diseases for healthy ageing: the vital public-private partnership project. Vaccine. (2020) 38(37):5896–904. 10.1016/j.vaccine.2020.07.005 32713682 PMC7378501

[34] Topol E. Deep medicine. New York: Basic Books (2019). 378 p.

[35] Cesari M Marzetti E Thiem U Perez-Zepeda MU Abellan Van Kan G Landi F, The geriatric management of frailty as paradigm of “the end of the disease era”. Eur J Intern Med (2016) 31:11–4. 10.1016/j.ejim.2016.03.005 26997416

[36] Kehler DS. Age-Related disease burden as a measure of population ageing. Lancet Public Health. (2019) 4(3):e123–e4. 10.1016/S2468-2667(19)30026-X 30851865

