# Supplementary material for: Infections and hospital bed-days among aging adults: A five-year retrospective study in a Belgian general hospital
Source: Front Med Technol. 2022 Sep 13;4:912469. doi: 10.3389/fmedt.2022.912469 (PMC9632861; doi:10.3389/fmedt.2022.912469)
Supplement: Supplementary file 1 [file Datasheet1.docx]

Supplementary Material

# The Infection Profile

## General statements

It should be noted that despite a high demand for lab identification of infection germs, only when the treating physician is convinced about the cause of infection will it be reported in the ICD-code in the registry. Therefore, expected completeness in precise diagnosis of germ specific infections is accurate under the conditions defined by the treating physician. Results presented hereunder should be interpreted with that assertion. They are however revealing some interesting findings.

## Infection in the geriatric ward

The disease description of the primary diagnosis of infection (n = 1,560, Category 1) in the geriatric ward was much more diverse compared with the pulmonology ward. There were 125 different ICD-9 codes used which was significantly higher as compared with the pulmonology ward (only 75 codes). But as in the pulmonology ward the dominance of respiratory infectious events was huge, with 34% for pneumonia, 9% for obstructive chronic bronchitis with acute exacerbation, 6% for bronchopneumonia, 4% for obstructive chronic bronchitis with acute bronchitis, 3% for bronchitis, and 2,5% for acute bronchitis. In the list of 10 most prevalent infectious diseases, we observed on number 3 erysipelas (6%), urine tract infection (5%), and intestinal infection (3%). The list had in addition infections in the intestinal organs, sepsis, esophagus, pancreas and gall bladder with candidiasis, campylobacter, clostridium, salmonella, and the like.

Secondary diagnosis (n=8,289, Category 2 & 4) had different appearances of diseases and frequencies as compared with primary diagnosis. The list had 413 different ICD-9-codes used which was again much larger than seen in the pulmonology ward (195 codes). Here, we observed a high dominance of urinary tract infections (22%), followed by Escherichia coli infections (15%), and a list of pulmonology infections including Streptococcus (3%), proteus (2%), gram-negative bacteria (2%), Klebsiella (2%), pseudomonas (2%), and tuberculosis (1%). Different from the pulmonology ward was a much higher frequency in septicemia caused by different pathogens (4% in total) and a much larger number of different organs that had an inflammation process ongoing like kidney, bladder, or skin.

The description of the nosocomial infections (n=2,899, Category 3 & 5) was quite in parallel with the secondary diagnosed infections regarding the most frequent appearances. There were 185 different ICD-9 codes used. Among the high frequency events we observed again the number one for urinary infections (19%). That shouldn’t be much of a big surprise given that urinary incontinence is a high frequency symptom among women living longer. There was more an equilibrium in the % of respiratory diseases (23% in total) and gastro-intestinal infections (21%) among the 10 most frequent events for this category of infections. We did not see the high frequency of tuberculosis in this group of patients/events as observed in the pulmonology ward.

For the non-infection group (n=11,353), we observed again marked differences in the profile of diseases to be treated in the geriatric ward as compared with the pulmonology group. There were in total 1,188 different ICD-9 codes used to describe the different medical activities performed in the geriatric ward for non-infection conditions. Four items characterized the group. Many of the aging adults were hospitalized for getting better care through revalidation (26%). More fractures (3%) were seen to be treated because of falling (geriatric profile) and the appearance of more dementia (5%). But there was a very diverse medical activity to be handled by the medical staff of which cardio (13%), traumata (10%), nerve diseases including Alzheimer (7%), revalidation (30%) were the bulk of medical care. Cancer was not so much present anymore in that age-group above 80 y old. This was also seen in the pulmonology and in the oncology ward.

An interesting observation was about comparing the group of No Infection during their stay (n=11,353) with the group that came in without infection but manifesting subsequently during their stay an infection (n=9,058, Category 4 & 5). The overall disease profile was quite different between the two groups related to cardio-vascular diseases (+3%) and fractures (+5%) for the group with infections. The latter was significantly more present amongst aging women who also suffered most of urinary tract infections. A logic reasoning behind those data could be that aging women, having many complaints about incontinence, may often wake up at night for that reason. With their old age, they are more likely to fall when standing up having difficulties to find immediately their exact equilibrium. Suffering from osteoporosis they will fracture their bones more often when they fall. The combination of fractures to be treated and being therefore immobile for a while, leads to urogenital infections, because of their suffering of urinary incontinence being the reason this infection was so frequently reported amongst them.

## Infection at the pulmonology ward

The disease description of the primary diagnosis of infection (n= 1,746; Category 1) in the pulmonology ward was conform the expecting findings. The ICD-9 codes indicated 38% of the events were obstructive chronic bronchitis with acute exacerbation, 24% being a pneumonia (no cause of origin), and 21% were obstructive chronic bronchitis with acute bronchitis. Then followed smaller % for bronchopneumonia (3%), acute bronchitis (3%), bronchitis (2%), pneumococcal and influenza pneumonia (2% each). Interesting to note was a 2% pleuritis caused by tuberculosis. The list was completed with a few cases of HIV, haemophiles influenza, and aspergillosis.

Secondary diagnosis of infection (n= 2,982, Category 2 & 4) had quite a different appearance of diseases and frequencies. Whereas for primary diagnosis we had with 10 ICD-9 codes a coverage of 95% of the total package of infections defined, in secondary diagnosed infections we had to use 195 different ICD-9 codes to cover the whole infection pool. There were a few particularities: a high frequency of inflammatory/infection events of the esophagus (14%), helicobacter pylori (9%), and 7% for tuberculosis. But other causes of infections were following at a much lower pace of less than 1% for hepatitis B, hepatitis C, candidiasis, herpes zoster, Escherichia coli, to name a few ones. Interesting to note was that the pulmonology ward had a high rate of lung cancer cases to treat often with radiotherapy which may be the reason to observe the high frequency of esophagitis and candidiasis infections in this pool of secondary diagnoses.

The description of the nosocomial infections (n= 608, Category 3 & 5) was again different from secondary and primary diagnosis where we found 54 different ICD-codes for those infections. We now suddenly observed the high appearance of tuberculosis that dominated the infection pool with 35% of the events followed by candidiasis (6%), acute exacerbations of obstructive chronic bronchitis (4%), aspergillosis (2%), helicobacter (2%), HIV (2%), and Escherichia coli (1%). Many other infection causes were at less than 1% each in this infection pool. Here also, there was an explanation why tuberculosis may have a high frequency of appearance. The ZNA group in Antwerp is one of the two reference centers of the disease management of tuberculosis in Belgium.

Finally, it should be noted that patients with their hospital stays, coming at the pulmonology ward with no infection (n=3,577), could be there for many diverse reasons going from the indication of obstructive sleep apnea (14%), cancer (18%), failure in heart disease (1.3%). Once, out of the infection pool of respiratory diseases, the pulmonology ward had a broad area of medical care to cover that encompassed as well mental health (14%), all kind of cardio-vascular diseases (12%), non-infectious lung diseases (15%), gastro-intestinal (10%), other supportive reasons (24%). There were in total 450 different ICD-9 codes used to describe the different activities performed in the pulmonology ward for non-infectious conditions.
